# Associations Between Consumption of Ultra-Processed Foods and Diet Quality Among Children and Adolescents

**DOI:** 10.3390/nu18020272

**Published:** 2026-01-14

**Authors:** Evgenia Petridi, Emmanuella Magriplis, Sotiria Kotopoulou, Niki Myrintzou, Evelina Charidemou, Elena Philippou, Antonis Zampelas

**Affiliations:** 1 Department of Life Sciences, School of Life and Health Sciences, University of Nicosia, Nicosia CY 2417, Cyprus; evipetridi@hotmail.com (E.P.);; 2 Department of Food Science and Human Nutrition, Agricultural University of Athens, 11855 Athens, Greece; emagriplis@aua.gr (E.M.); nmyrintzou@aua.gr (N.M.); 3 Hellenic Food Authority, 11526 Athens, Greece; skotopoulou@efet.gr; 4 Department of Nutritional Sciences, King’s College London, London SE1 9NH, UK

**Keywords:** ultra-processed foods, diet quality, childhood, adolescence, non-communicable chronic diseases

## Abstract

**Background**: Ultra-processed foods (UPFs) have emerged as a critical component of diet quality, yet data on the associations between UPF and nutrient intakes remain limited. This study aimed to evaluate nutrient consumption in relation to UPF intake and adherence to international dietary guidelines for non-communicable disease (NCD) prevention. **Methods**: Data from 469 individuals aged 2–18 years enrolled in the Hellenic National Nutrition and Health Survey (HNNHS) were analyzed. Intakes were assessed using two 24 h recalls, and foods were classified according to the NOVA system. Participants were categorized by UPF energy intake tertiles. Nutrient adequacy was assessed using Nordic Nutrition Recommendations, European Society of Cardiology guidelines for macronutrients, and the Institute of Medicine’s Estimated Average Requirements and Adequate Intake values for micronutrients. **Results**: Children in the highest UPF tertile had significantly higher intakes of energy, carbohydrates, added sugars, saturated fats, polyunsaturated fats, and cholesterol, but lower intakes of protein compared to those in the lowest tertile. Fiber intake remained inadequate across all tertiles, with no significant differences. Regarding adherence to NCD prevention guidelines, children in the 3rd UPF tertile had a 2.3 times higher prevalence ratio for exceeding added sugar recommendations, while their protein intake prevalence ratio was 0.8 times lower. For micronutrients, the highest UPF tertile showed significantly elevated intakes of vitamins E, B_1_, folate, calcium, iron, copper, and sodium, but lower potassium intake compared to the lowest tertile. **Conclusions**: Our results underscore the need for effective public health strategies to improve diet quality in children and adolescents and prevent diet-related NCDs.

## 1. Introduction

Chronic non-communicable diseases (NCDs) can arise from a variety of causes, with an unhealthy diet being one of the most important modifiable risk factors. Although adulthood is the most common time period at which clinical manifestations of chronic diseases are observed, exposure to risk factors earlier in life increases the risk due to potential long-term accumulation [1]. It is also increasingly accepted that a dietary pattern is not only determined by its content in specific nutrients or food items, but also by potential hazards that may accumulate during processing. The extent and type of food processing have recently gained prominence and are being investigated as to their potential role in diet-related NCDs [2]. 

Ultra-processed foods (UPFs) are industrial formulations that use several ingredients, little or no whole foods, undergo a series of processes, and are combined with sophisticated use of additives to make them long-lasting and extremely palatable. They are ready-to-eat or ready-to-heat, requiring little or no culinary preparation, and thus are easily accessible and convenient [3,4]. UPF consumption has been linked to several indicators of poor nutritional quality. They are typically energy dense, high in refined starch, added sugar, saturated fat, trans-fat, and sodium, and low in fiber, protein, vitamins, and minerals [3]. Consequently, the level of processing has emerged as an important new dimension of diet quality, reflecting a qualitative aspect of food that is frequently overlooked in traditional nutrient-focused measurements [5]. However, the application of the NOVA system is not without debate. Critics argue that the broad categorization may overlook nutritional heterogeneity within the ‘ultra-processed’ group, potentially equating nutrient-dense, fortified foods (such as certain breakfast cereals or enriched breads) with nutrient-poor items like sugary beverages [6]. This lack of granularity can complicate the assessment of diet quality, particularly regarding micronutrient intake, where fortification plays a significant role.

There is little evidence of a link between UPF consumption and nutrient composition or its health impact in children [7,8]. An analysis of 10 cycles of the National Health and Nutrition Examination Survey (NHANES) conducted in children and adolescents aged 2–19 years showed that the majority of their daily energy intake comes from UPF consumption, with parallel high intakes of carbohydrates, added sugars, total fats and polyunsaturated fats, and low intakes of protein, fiber, calcium, magnesium, potassium, zinc, vitamins A, C, and D, and folate [9]. The Canadian Community Health Survey also showed that more than half of the daily energy intake of children and adolescents came from UPFs [3]. Moreover, a cross-sectional study in the UK in children aged 1.5 years and older reported that UPF consumption was positively associated with energy, carbohydrates, free sugars, total fats, saturated fats, and sodium and inversely associated with protein, fiber, and potassium intakes [4]. In addition, a meta-analysis of nationally representative surveys showed that an increase in UPF consumption led to an increase in the intake of energy, total and saturated fat, and free sugars, as well as a reduction in intakes of protein, fiber, vitamins A, C, D, E, B12, niacin, potassium, magnesium, and zinc [10]. 

Using the controversial NOVA classification, consumption of UPF has been associated with disease risk not only in adults but also in children and adolescents. In particular, in a prospective study, children with a higher consumption of processed foods and ultra-processed foods and a higher Children’s Dietary Inflammatory Index showed an increase in body fat during adolescence regardless of Body Mass Index (BMI) [11]. In another study in the Mediterranean region [12], higher intake of UPF was associated with obesity. In the same study, children who ate more frequently out of the home and a higher number of meals were also more likely to consume unhealthier UPFs. In another study by Calcaterra and co-workers, high UPF intake was associated with increased adiposity, metabolic dysregulation, greater cardiometabolic risk, and in children with obesity, a higher prevalence of functional gastrointestinal disorders was reported compared to the normal-weight controls [13]. Finally, in a pilot case-control study, children with ulcerative colitis were found to have higher dietary intake of UPFs than their healthy controls [14]. 

On the other hand, in a European study, although a decline in diet quality was observed with increasing UPF intake, no association between UPF consumption and Metabolic Syndrome or its individual components was reported [15]. These results are in accordance with the findings of our study, which found no association between UPF consumption and obesity in children and adolescents [16]. 

In Greece, a high prevalence of childhood obesity is reported [17,18], which is associated with dietary, lifestyle, and family environment factors [19]. In addition, children’s and adolescents’ diet was found to be high not only in sugars [20], and sodium [21] but also in nitrates and nitrites [22]. Furthermore, the Greek food environment presents a unique landscape regarding micronutrient availability in packaged foods. Recent analysis of the Greek Branded Food Composition Database (HelTH) indicates that approximately 11% of branded products carry micronutrient claims, with the majority (64%) of these products achieving these claims through artificial fortification rather than natural content [6]. This prevalence of market-driven fortification in popular ultra-processed categories, such as sweet grain products, may significantly influence the micronutrient intake profile of Greek children. However, the association of UPF consumption with diet quality has not yet been studied in Greek children.

Therefore, the objective of this study was first to assess the association between UPF consumption and diet quality in children and adolescents using macro- and micronutrient consumption as proxy measures of dietary quality and, second, to assess how UPF consumption affects their adherence to recommended international guidelines established for the prevention of NCDs.

## 2. Materials and Methods

### 2.1. Population Sample and Study Design

Data were derived from the Hellenic National Nutrition and Health Survey (HNNHS), a population-based study conducted between September 2013 and May 2015, designed to assess the health and nutritional status of children and adults. Sampling and design details have been published elsewhere [19]. Data on anthropometric, sociodemographic, and lifestyle characteristics were collected during an in-person interview at the participant’s residence by trained interviewers using the Computer-Assisted Personal Interview (CAPI) method. For participants under the age of 18, parents or guardians provided written informed consent; adolescents provided additional written consent. The study was approved by the Ethics Committee of the Department of Food Science and Human Nutrition of the Agricultural University of Athens in 2013. The study was also approved by the Hellenic Data Protection Authority. It was conducted in accordance with the 1964 Declaration of Helsinki and its later amendments or comparable ethical standards.

Of the 478 children eligible for the analyses, 9 did not consume foods from the NOVA 4 food group. Thus, the final sample consisted of 469 children with complete information.

### 2.2. Data Collection

#### Dietary Assessment

Detailed information on dietary intake was collected through 24 h dietary recalls by trained interviewers using the Automated Multi-pass Method (AMPM) [23]. All children provided at least one recall, which was conducted in person. Specifically, of the 478 participants, 77.6% (n = 371) completed two recalls and 22.4% (n = 107) completed one recall. Weekend days were represented in 47.7% (n = 228) of participants (29.8% of all recalls). For children < 12 years, primary guardians were interviewed, while adolescents ≥ 12 years completed the dietary recall independently, with the help of their guardian. Age-specific food atlases and common household measurements (e.g., glasses, cups, spoon sizes) were used to estimate precise portion sizes. More child-specific information can be found in [24,25]. 

The Nutrition Data System for Research (NDSR), developed by the University of Minnesota, and the Greek food composition tables for traditional Greek recipes (e.g., baklavas) [26] were used to calculate total energy and macro- and micronutrient intake values. Furthermore, all foods identified from the 24 h recalls were then classified using the NOVA classification system [2]. (Food categorization per NOVA group has been published in detail, while the main foods placed in each NOVA can be viewed in the Supplementary Materials (Supplementary Table S1). To ensure accurate classification under the NOVA system, a specific protocol was employed for mixed dishes and composite foods. Traditional Greek recipes and homemade dishes (e.g., baklavas, pastitsio, and spinach pies) were disaggregated into their raw ingredients using Greek food composition tables. Each constituent ingredient was then individually classified into the appropriate NOVA group (e.g., flour and nuts to NOVA 1; olive oil and sugar to NOVA 2; and artisanal phyllo dough to NOVA 3). In contrast, mass-produced, pre-packaged mixed dishes (e.g., frozen ready-to-heat pies, instant soups, and reconstituted meat products) were not disaggregated but were classified as single items under the NOVA 4 category (Ultra-Processed Foods). This distinction relies on the presence of industrial additives (e.g., emulsifiers, colors, and flavor enhancers) characteristic of ultra-processing. Examples include flavored yogurts, mass-produced packaged breads, breakfast ‘cereals’, carbonated soft drinks, reconstituted meat products, and packaged instant soups. A detailed description of the NOVA classification can be found elsewhere [27,28,29,30]. Two authors independently reviewed each item’s classification.

### 2.3. Nutritional Assessment

**Figure 1:**
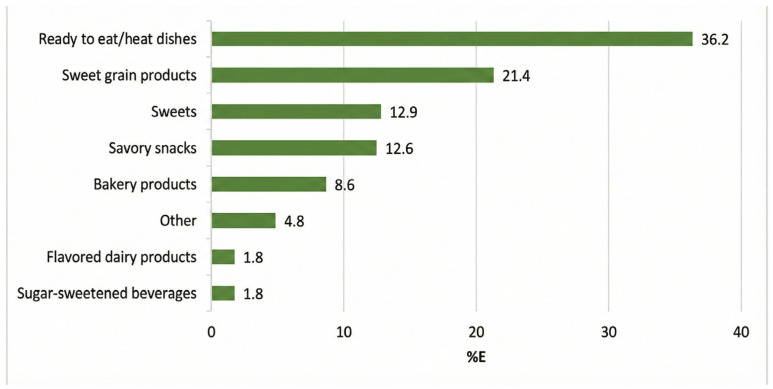
NOVA 4 subgroup contribution to total energy intake (total children) [16].

The total daily energy intake (TEI) of NOVA 4 classified foods was calculated by summing the energy content of each food item, following which, the average intake per child per day was estimated. The same process was followed for all macro- and micronutrients. The percentage contribution of total energy intake for NOVA 4, along with the main food contributors, was then calculated for the total sample. This was calculated by dividing the TEI of each NOVA food by the total daily energy intake reported by each child or guardian and then multiplying it by 100 [31]. Details have been previously published [16], but a graph is provided as an adapted figure for total children (Figure 1). In summary, ready-to-eat/heat dishes (36.2%), sweet grain products (21.4%), savory snacks (15.4%), and sweets (12.9%) are the primary drivers of UPF intake in this population. Population tertiles were then calculated for each of the assessed nutrients.

The nutritional indicators examined included total carbohydrates, added sugars, proteins, fiber, total fat, dietary fatty acids (saturated and polyunsaturated), cholesterol, fiber, and a total of 21 vitamins and minerals. All macronutrient intakes were expressed as a percentage of kcal per total intake for each child. Dietary cholesterol and fiber intakes were expressed in mg and grams, respectively. Intakes above the recommended dietary nutrient goals for the prevention of chronic diseases were derived as per the Nordic Nutrition Recommendations [32] and ESC Scientific Document Group [33] (Mach et al., 2020) (Table 1).

The corresponding intake cut-off for vitamins and minerals for children and adolescents was the Estimated Average Requirement (EAR) set by the Institute of Medicine (IOM). For micronutrients such as potassium, pantothenic acid, and vitamin K, the proportion below the Acceptable Intake (AI) was used instead, since there are no EARs for these nutrients. The cut-off of >2300 mg/day was used for sodium consumption, since it is the maximum accepted level for adolescents.

### 2.4. Other Covariates

A general questionnaire was used to collect details on socioeconomic data such as age, sex, screen time, parental employment status, and parental educational level. Screen time was defined as the weekly average of time spent in front of any type of screen, excluding active gaming. Total screen time was classified using the upper recommended level of 2 h per day [34]. The parental socioeconomic status was determined by their educational level (elementary school, middle school, or higher level of education) and employment status (employed, unemployed, or pension). Body Mass Index (BMI) was used to evaluate the children’s weight status and was calculated by dividing weight in kg by height in meters squared (kg/m^2^). BMI status was then classified using the extended International Obesity Task Force (IOTF) tables [35].

### 2.5. Statistical Analyses

Participants were categorized into tertiles based on their percentage contribution of UPFs to total energy intake (NOVA-4). Descriptive statistics are presented as means (SD) for continuous variables following normal distribution, medians with interquartile ranges (IQR) for variables with skewed distributions, and as frequencies with percentages for categorical variables. The anthropometric and sociodemographic characteristics across tertiles were estimated.

To examine associations between UPF tertiles and macronutrient intakes, generalized linear models (GLMs) with a gamma distribution and log link function were employed, accounting for the right-skewed nature of dietary intake data. The exponentiated coefficients represent the multiplicative change in mean nutrient intake across UPF tertiles.

Models were adjusted for potential confounders, including sex, age, and total screen time (hours/day). To test for linear trends across UPF tertiles, p-for-trend was calculated by assigning the median UPF intake value within each tertile to all participants in that tertile and modeling this as a continuous variable in the regression models. This approach accounts for the actual distribution of UPF intake within tertiles rather than assuming equal spacing between categories.

For vitamins and minerals that resulted in a significant intake difference by level of NOVA 4 tertiles, binary indicators were developed based on upper recommended level (macronutrients) and EAR for micronutrients. Modified Poisson regression with robust variance estimation was used to estimate prevalence ratios (PRs) and 95% confidence intervals (CIs) for exceeding or not meeting these guidelines across UPF tertiles, with the lowest tertile serving as the reference group. Models were adjusted for sex, age, area of residence, and total screen time.

Outcomes are presented according to corresponding 95% confidence intervals. Statistical significance was set at alpha 5% (*p* < 0.05). All statistical analyses were carried out using the statistical software package STATA 19.0 (Stata Corp LLC., College Station, TX, USA).

## 3. Results

Based on the energy contribution from UPFs, participants were divided into three tertiles. Table 2 depicts the distribution of anthropometric and sociodemographic variables for the entire population as well as across UPF consumption tertiles. Significant between-group differences were found for age (*p* = 0.003) and for primary guardian education level (*p* = 0.05). It is noteworthy that total screen time was higher with increasing UPF tertiles (*p* = 0.003). No other significant differences were found.

Table 3 depicts the median content of macronutrient dietary cholesterol and dietary fiber intakes in relation to energy intake quintiles per population tertile of UPF intake. The table also depicts the exponentiated beta coefficient, expressed as mean ratios (SE), and shows how dietary intake patterns differ across tertiles of UPF consumption. The median energy intake increased from 1416 kcal/day (Q1) to 1920 kcal/day (Q3), while after adjusting for covariates, mean energy intake was 1.30 times higher in Q3 vs. Q1. A significant increase was also found for carbohydrates (exponentiated β = 1.08, SE = 0.03, *p* = 0.002) and added sugars, which increased 1.7 times (exponentiated β = 1.72, SE = 0.12, *p* < 0.001). A higher intake was found for SFA, PUFA, and cholesterol for children consuming more UPF (3rd tertile) compared to the lowest (1st tertile) (*p* for all < 0.001). In terms of protein, an inverse association was found with 20% less protein intake consumed in the 3rd UPF tertile compared to the 1st (0.8 (0.03, *p* < 0.001)). A linear increasing trend was found for all mentioned. It is also noteworthy that children had low fiber intakes (11–13 g/day vs. ≥25 g recommended) across all tertiles, with no significant differences found with increasing UPF intake.

Table 4 presents the median content of micronutrients in the overall diet across tertiles of the children and adolescents’ UPF consumption, with a derived assessment of intake by UPF tertile. Children in the highest UPF tertile had significantly higher intakes of vitamin E (*p* = 0.001), vitamin B1 (*p* = 0.002), folate (0.05), calcium (0.000), iron (0.02), copper (0.003), and sodium (0.004), and lower intakes of potassium (*p* = 0.02) compared to the lowest tertile. Specifically, after adjusting for sex, age, and screen time, those in the highest tertile were 1.79, 1.09, 1.10, and 1.24 times more likely to have higher vitamin E, vitamin B_1_, iron, and folate intakes, respectively. The same trend, although not statistically significant, was also found for calcium and copper. An increasing trend was also found for sodium (1.15 times more likely to have higher intakes), whereas children in the highest UPF category consumed 64% less potassium (*p* for all < 0.02). A linear trend was found for intakes of vitamin E, vitamin B_1_, folate, calcium, iron, copper, sodium, and potassium (*p* for all < 0.02).

**Figure 2:**
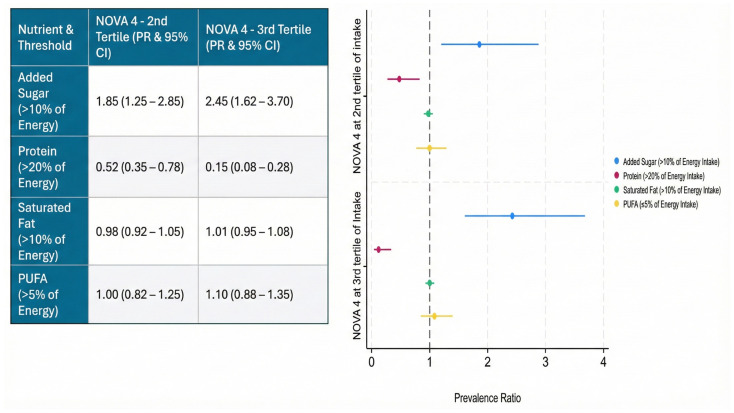
Association between NOVA 4 levels and macronutrient intake (as per dietary guidelines). Prevalence ratios of children and adolescents consuming specific macronutrients beyond recommendations. Added sugar > 10% of total energy, total protein > 20% of total energy, saturated fatty acids (SFA) > 10% of total energy, polyunsaturated fatty acids (PUFA) < 5% of total energy and cholesterol > 300 mg/day. Level of significance set at alpha 5%. Results follow Poisson regression (robust) analysis adjusted for age, sex, area of residence, and total screen hours. The 1st tertile (lowest UPF consumption) served as the reference group (PR = 1.00) and is therefore not plotted.

Figure 2 shows the association between NOVA 4 levels and macronutrient intakes in comparison with dietary guidelines for NCD prevention. It is noteworthy that compared to the 1st UPF tertile, the prevalence ratio of intakes of added sugars above the upper recommended limit for children int the 2nd and 3rd UPF intake tertiles, was 1.8 and 2.3 times higher, respectively. Moreover, the prevalence ratio for dietary protein intake was 0.2 and 0.8 times lower, respectively. No associations were observed for saturated fat and PUFA intakes.

**Figure 3:**
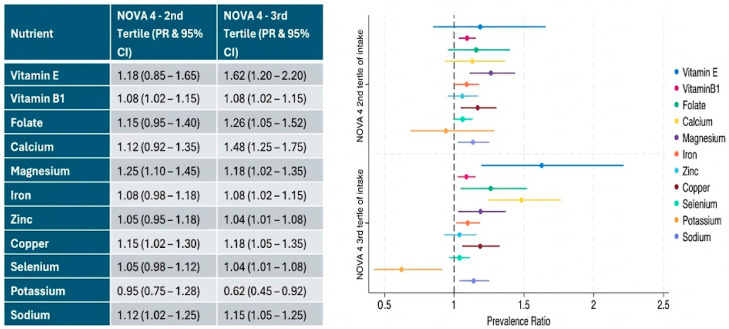
Association between NOVA 4 levels and micronutrient intake (above average requirements). Prevalence ratios of children and adolescents consuming micronutrients above recommendations. Based on EAR values or AI values when EAR was not available (IoM). Results follow Poisson regression (robust) analysis for levels of consumption in relation to average recommendations (either EAR or AI) adjusted for age, sex, and total screen hours. Level of significance set at alpha 5%. The 1st tertile (lowest UPF consumption) served as the reference group (PR = 1.00) and is therefore not plotted.

Associations between UPF intake levels and micronutrient intake (above EAR or AI) are shown in Figure 3. In particular, a significant increase in vitamin or mineral intake compared to EAR or AI recommendations was found for vitamins B_1_, magnesium, copper, and sodium, by increasing UPF intake, whereas vitamin E, folate, calcium, and iron (borderline) were higher only in children categorized at the 3rd tertile of UPF consumption. Potassium, however, was significantly lower in children in the 3rd UPF tertile, compared to children in the 1st tertile.

## 4. Discussion

The main finding of our study is that children in the highest UPF tertile derived approximately three-quarters of their total energy intake from ultra-processed foods and had significantly different macro- and micronutrients than those in the lowest tertile (19.6% of energy from UPF). Specifically, among macronutrients, children in the highest UPF tertiles had higher intakes of total energy, carbohydrates, added sugars, SFA, sodium, and cholesterol, of which carbohydrates, added sugars, and sodium exceeded nutritional recommendations. Conversely, these children also had significantly lower intakes of protein. In terms of micronutrients, although for many vitamins a significantly higher intake than the EAR was observed in children at the highest UPF tertile, potassium consumption was significantly lower in 40% of children. Conversely, sodium intake exceeded the maximum recommended intake for adolescents (the highest recommendation among children), including in the 2nd UPF tertile, compared to the lowest. Notably, dietary fiber intake was inadequate across all UPF tertiles, regardless of the consumption level. Additionally, UPF consumption as a percentage of total daily energy intake was significantly associated with age, body weight, screen time, and primary guardian education level.

Recent studies reported an association between UPF intake and an unhealthier dietary content [3,10,36,37,38]. In particular, Araya et al. showed that preschoolers in the highest UPF tertile consumed more energy, carbohydrates, total sugars, saturated and monounsaturated fats, and vitamin D than those in the lowest UPF tertile, while also consuming less protein, polyunsaturated fats, dietary fiber, zinc, vitamin A, and sodium [36]. In addition, Moubarac et al. [3] showed that the dietary contribution of UPFs was positively correlated with energy density, carbohydrates, free sugars, and total and saturated fats, and inversely correlated with the intake of protein and fiber; vitamins A, C, D, B_6_ and B_12_; niacin, thiamine, and riboflavin; and zinc, iron, magnesium, calcium, phosphorus, and potassium [3]. In a relatively recent meta-analysis it was reported that higher consumption of UPFs was associated with a global trend toward poorer nutritional quality, specifically a greater intake of sugars, total fats, and saturated fats—increasing the risk of chronic diseases in adulthood—and a lower intake of fiber, protein, potassium, zinc, and magnesium, as well as vitamins A, C, D, E, B12, and niacin [10]. Romeiro et al., in their study, observed that the consumption of UPF increased the densities of carbohydrates, free sugars, saturated fat, and Na and decreased the densities of proteins, fiber, and potassium in adolescents [37]. Lastly, in a study from Argentina, a positive association between UPF intake and free sugars and total saturated and trans-fatty acids, and a negative association with fiber and protein were observed [38]. 

Unexpectedly, our study showed an increased intake of several vitamins and minerals in those in the highest UPF tertile, which is in contrast with the results of the studies mentioned above. This may be related to the different foods that contribute to the UPF intakes in different countries. In HNNHS, four major food groups were found to contribute > 85% of total UPF intake: ready-to-eat/heat dishes (36.2%), sweet grain products (21.4%), savory snacks (15.4%), and sweets (12.9%), and these provided 86% of the total UPF intake [16]. Sweet grain products are major sources of vitamin and mineral fortification, which may explain the increased intake observed in the 3rd UPF tertile in our study. It is noteworthy that in Brazil, market-driven fortification is present in 27% of packaged foods targeting children, with vitamins and minerals commonly added as a marketing strategy [39]. Analysis of the Greek Branded Food Composition Database (HelTH) found that 11% of products in Greece are marketed as “high in” or a “source of” micronutrients. However, only 36% of these products are natural sources of the claimed micronutrients, meaning that the majority (64%) achieve these claims through fortification [6]. In our study, we also observed low intakes of dietary fiber and potassium, indicating that the diet of children and adolescents is poor in fruits and vegetables, and consequently, these foods could not contribute to the increased vitamin and mineral intakes. This mirrors market-driven fortification strategies seen in Brazil, where 27% of child-targeted packaged foods are fortified. Consequently, children in the highest UPF tertile may technically meet EARs for vitamins B_1_, folate, and iron through industrial additives, despite consuming a diet of poor overall quality. Therefore, there is a significant controversy about using UPFs as fortification vehicles since on the one hand, they could claim to reduce micronutrient deficiencies but, on the other hand, they contribute to high intakes of SFA, added sugar, and sodium. Therefore, it can be justified that their benefits outweigh the health risks [40]. 

Another noteworthy finding of our research is that, even in the lowest tertile of UPF consumption, the majority of children did not meet the Nordic recommended cut-offs for NCD prevention for added sugars. Specifically, in the highest tertile of UPF intake, about 40% of participants surpassed the Nordic upper limit for added sugars (<10%). Similar results were found in a study with 9317 US participants who were at least one year old. Compared to 26.4% in the lowest quintile, 82.1% of Americans in the highest quintile consumed more added sugar than the recommended 10% energy limit [41]. 

Finally, our study found that sodium intake exceeded the recommended intake in all tertiles [42]. In the UK, similar observations were made [4]. However, studies from Chile [43], Canada [3], and the United States [44] did not show a positive association between sodium intake and the dietary share of UPFs. This might be because different countries consume different kinds of ultra-processed foods. For example, Greece tends to consume more salty items than the USA and Canada, which tend to consume more sweetened products [3,44]. 

It should be noted that socioeconomic status impacts not only the amount but also the type of UPF consumed by adolescents, underscoring the importance of this factor when designing interventions [45]. In addition, it has also been reported that children and adolescents from families of low socioeconomic status showed higher prevalence of food insecurity and lower dietary quality, characterized by lower adherence to the Mediterranean diet and higher consumption of UPF [46]. 

This study has several strengths. To our knowledge, this is the first study to evaluate dietary UPF intake and its main contribution to energy and macro- and micronutrient intakes among Greek children and adolescents. As per the study results, this is an important area to examine, since the effect of UPFs on various nutrients and potentially on health possibly varies by type and amount of food group consumed. However, there are limitations. We acknowledge the ongoing debate regarding the NOVA classification system, particularly the potential for misclassifying nutrient-dense processed foods. To mitigate this, we employed a strict disaggregation protocol for traditional mixed dishes, classifying only industrial formulations as UPF (NOVA 4). Additionally, the cross-sectional design limits inference on the time sequence of the association between UPF consumption and diet quality. It is also critical to consider any potential NOVA system food misclassifications.

There are some limitations of our study. The cross-sectional design limits inference on the time sequence of the association between UPF consumption and diet quality. It is also critical to consider any potential NOVA system food misclassifications. Finally, even though the HNNHS collects specific information on food processing (such as meal locations and product brands), this information was not consistently gathered for all food products, which could lead to an underestimation of UPF intake.

## 5. Conclusions

In conclusion, our findings support the idea that greater dietary intake of UPF demonstrates a controversy in the quality of children’s and adolescents’ diets, since on the one hand it was characterized by a higher dietary content of NCD-promoting nutrients, added sugars, saturated fat, cholesterol, and sodium and was lower in protein and potassium, but on the other hand it was higher in several vitamins and minerals. These observations highlight the necessity of a distinction between healthy and unhealthy UPFs and the need for more research to be carried out to examine these discrepancies in greater depth. Public health strategies to decrease UPF intake indiscriminately may not be the correct direction.

## Figures and Tables

**Table 1:** Macronutrient dietary guidelines are used to assess diet quality.

Macronutrient	Intake Cut-Offs
Carbohydrates *	60% of total energy
Added sugars *	10% of total energy
Total fats *	40% of total energy
Saturated fats *	10% of total energy
PUFA *	<5% of total energy
Dietary fiber *	<25 g/day
Cholesterol ^+^	<300 mg/day
Sodium *	<2300 mg/day

* [32], ^+^ [33].

**Table 2:** Distribution of anthropometric and sociodemographic characteristics for the whole population and across tertiles of energy contribution of ultra-processed foods.

	Median Relative Intake of UPFs (% of Total Energy Intake)				
	Total 38.6% (25.5, 54.6)	Tertiles of the Contribution of UPFs to Total Energy Intake			
		Q1 19.6% (13.4, 25.5)	Q2 38.6% (33.9, 44.4)	Q3 60.8% (54.8, 72.6)	*p*-Value
**n, (%)**	469	157 (33.4)	156 (33.3)	156 (33.3)	
**Age years, median (IQR)**	9 (6, 13)	7 (3, 14)	9 (6, 13)	9.5 (6.5, 14)	0.003
**ΒΜΙ, kg/m^2^, mean, (SD)**	18.8 (4)	18.2 (3.8)	19.1 (4.1)	19 (4.1)	0.493
**Age group, n, (%)**					0.681
Children (2–11 years)	309 (65.9)	107 (22.8)	103 (22)	99 (21.1)	
Adolescents (12–18 years)	160 (34.1)	50 (10.7)	53 (11.3)	57 (12.1)	
**Sex n, (%)**					0.754
Male	237 (50.5)	83 (17.7)	78 (16.6)	76 (16.2)	
Female	232 (49.5)	74 (15.8)	78 (16.6)	80 (17.1)	
**Weight Status n, (%)**					0.40
Normal weight	323 (74.4)	110 (25.4)	103 (23.7)	110 (25.3)	
Overweight/obese	111 (25.6)	31 (7.1)	42 (9.7)	38 (8.8)	
**Total screen time (hours), median (IQR)**	2.2 (1.25, 3.5)	2 (1, 3)	2.2 (1, 3.5)	2.5 (1.8, 3.7)	0.006
**Primary guardian education level, n (%)**					0.05
≤6 years	12 (3.8)	3 (1.0)	8 (2.6)	1 (0.3)	
>6–12 years	123 (39.3)	42 (13.4)	35 (11.2)	46 (14.7)	
≥12 years	178 (56.9)	47 (15)	67 (21.4)	64 (20.4)	
**Primary guardian professional status, n (%)**					0.441
Employed	215 (70.0)	59 (19.2)	81 (26.4)	75 (24.4)	
Unemployed/ Homeworkers	77 (25.1)	28 (9.1)	21 (6.8)	28 (9.1)	
Pension	15 (4.9)	5 (1.6)	6 (2.0)	4 (1.3)	

*p* < 0.05; one-way ANOVA test for normally distributed values and Kruskal–Wallis test for skewed numerical variables (three-group comparison); chi-square test for categorical variables, IQR: interquartile range.

**Table 3:** Median macronutrient, dietary cholesterol, and dietary fiber intakes in relation to intakes by tertiles of ultra-processed foods as per mean energy intake.

Indicator ^a^	Overall Diet	Intakes by Tertiles of Ultra-Processed Foods as Per Mean Energy Intake			Adjusted GLM Model ^b^ (Mean Ratios ^c^, (SE))				
					Q2 vs. Q1 (SE)	*p*-Value	Q3 vs. Q1 (SE)	*p*-Value	*p*-for Trend
	Total	Q1 19.6 (13.4, 25.5)	Q2 38.6 (33.9, 44.4)	Q3 60.8 (54.7, 72.6)					
**Energy (kcal/day)**	1655 (1299, 2185)	1416 (1127, 1776)	1697 (1366, 2148)	1920 (1477, 2426)	1.12 (0.06)	0.022	1.30 (0.07)	0.000	0.000
**CHO** **(% energy)**	43.5 (38.3, 49.5)	42.6 (36, 48.3)	45.0 (38.9, 50.2)	43.7 (38.6, 49.2)	1.08 (0.03)	0.002	1.08 (0.03)	0.002	0.003
**Added sugars** **(% energy)**	6.8 (3.8, 10.8)	4.5 (2.3, 8.8)	7.7 (5.1, 10.8)	8.1 (4.9, 13)	1.42 (0.12)	0.000	1.72 (0.15)	0.000	0.000
**Protein (% energy)**	15.2 (13, 17.7)	15.9 (14, 19.4)	15.1 (12.9, 17.5)	14.6 (11.7, 16.2)	0.91 (0.03)	0.003	0.80 (0.03)	0.000	0.000
**Fat** **(% energy)**	41.3 (36.2, 47.5)	41.7 (36.1, 47.1)	40.1 (35.9, 45.9)	42.4 (36.9, 48.4)	0.96 (0.02)	0.082	1.00 (0.02)	0.9	0.953
**Cholesterol (mg/d)**	378 (229, 562)	385 (232, 609)	353 (222, 521)	385 (225, 543)	1.08 (0.03)	0.002	1.08 (0.03)	0.002	
**SFA** **(% energy)**	15.4 (12.7, 18.4)	14.8 (11.9, 18.2)	15.3 (12.6, 18)	16 (13.4, 18.8)	1.01 (0.03)	0.718	1.08 (0.04)	0.015	0.014
**PUFA** **(% energy)**	4.9 (4.1, 6.5)	4.9 (4.2, 6.2)	4.9 (4.2, 6.1)	5.2 (3.9, 7.6)	1.03 (0.06)	0.683	1.18 (0.07)	0.07	0.006
**Dietary fiber (g/d)**	12.3 (8.6, 17.1)	11.3 (7.6, 16.3)	13.1 (9.9, 17.9)	12.3 (9, 17.2)	1.09 (0.08)	0.198	1.05 (0.07)	0.494	0.518

^a^ All values refer to medians; IQR: interquartile range; ^b^ adjusted for age (years), sex, screen time (hrs), parents’ educational level (≤6 years, >6–12 years, ≥12 years), and NOVA 4 tertiles; ^c^ exponentiated b-coefficients; *p* ≤ 0.05 for linear trend across tertiles of ultra-processed food consumption.

**Table 4:** Median content of micronutrients in the overall diet across tertiles of ultra-processed food consumption in accordance with IOM’s recommendations in children and adolescents.

Indicator ^a^	Overall Diet	Intakes by Tertiles of Ultra-Processed Foods as Per Mean Energy Intake ^a^			b-Coefficient (SE) Q2 vs. Q1 ^b^	*p*-Value	b-Coefficient (SE) Q3 vs. Q1 ^b^	*p*-Value	*p*-for Trend
	Total	Q1 19.6 (13.4, 25.5)	Q2 38.6 (33.9, 44.4)	Q3 60.8 (54.7, 72.6)					
**Vitamin A** **(μg/d)**	1090.6 (718.5, 1604.6)	899.9 (637.1, 1545.4)	1106.7 (756.7, 1586)	1195.4 (785, 1780.8)	0.01 (0.03)	0.057	0.03 (0.06)	0.059	0.099
**Vitamin D (μg/d)**	5.2 (3, 7.3)	5.5 (3.8, 7.9)	4.9 (3, 6.8)	4.8 (2.5, 7.2)	0.6 (0.28)	0.271	1.25 (0.57)	0.619	0.489
**Vitamin E (mg/d)**	5.8 (3.9, 9.5)	5.1 (3.4, 8)	6.1 (4.3, 9.3)	6.6 (4.1, 12.2)	1.11 (0.19)	0.543	1.79 (0.31)	0.001	0.001
**Vitamin K (μg/d)**	18.9 (11.3, 33.6)	15.6 (9.7, 29.5)	20.1 (12.5, 33)	20.8 (12.5, 40.4)	0.78 (0.31)	0.535	1.33 (0.5)	0.451	0.342
**Vitamin C (mg/d)**	56.6 (28, 99.4)	61.5 (30.6, 102.6)	60.5 (28.5, 99.3)	46.8 (27, 94.5)	0.95 (0.08)	0.516	0.86 (0.07)	0.058	0.058
**Vitamin B_1_ (mg/d)**	1.4 (1.1, 1.9)	1.2 (0.9, 1.7)	1.5 (1.1, 1.9)	1.6 (1.2, 2.3)	1.09 (0.03)	0.001	1.09 (0.03)	0.002	0.002
**Vitamin B2 (mg/d)**	1.7 (1.3, 2.2)	1.7 (1.2, 2.1)	1.7 (1.3, 2.2)	1.8 (1.4, 2.6)	0.99 (0.02)	0.727	1 (0.02)	0.899	0.876
**Niacin** **(mg/d)**	12.9 (8.5, 20.3)	10.8 (6.7, 18.4)	13.2 (9.4, 20.2)	14.3 (9.7, 22.9)	1.12 (0.07)	0.06	1.09 (0.07)	0.146	0.163
**Pantothenic acid** **(mg/d)**	3.3 (2.5, 4.3)	3.3 (2.5, 4.3)	3.2 (2.5, 4.1)	3.3 (2.4, 4.4)	1.06 (0.15)	0.689	0.99 (0.14)	0.926	0.914
**Vitamin B_6_ (mg/d)**	1.4 (1, 2.2)	1.5 (1, 2)	1.4 (1.1, 2.2)	1.4 (0.9, 2.2)	1.04 (0.04)	0.269	0.97 (0.04)	0.438	0.405
**Folate** **(μg/d)**	232.9 (154, 335.1)	200.9 (124, 312.3)	248 (165, 326.9)	249.6 (176.8, 407)	1.17 (0.12)	0.141	1.24 (0.13)	0.05	0.054
**Vitamin B_12_ (μg/d)**	3.1 (2, 4.6)	3.1 (2, 4.4)	3 (2, 4.4)	3.1 (1.9, 4.9)	1.03 (0.04)	0.529	0.99 (0.04)	0.827	0.804
**Calcium** **(mg/d)**	966.8 (691.6, 1270.7)	879.4 (649, 1088.2)	935.4 (693.2, 1237.8)	1096.9 (805.6, 1476.9)	1.16 (0.14)	0.225	1.59 (0.2)	0.000	0.000
**Phosphorus (mg/d)**	1032.8 (807.8, 1342.1)	967.7 (779.4, 1279.5)	1016.6 (805.1, 1311.1)	1132.9 (850.6, 1450.6)	0.32 (0.2)	0.072	0.31 (0.2)	0.073	0.086
**Magnesium (mg/d)**	203 (161.2, 261.2)	186.2 (147.4, 241.6)	208.2 (170.7, 269.7)	203.1 (163.4, 277.2)	1.38 (0.15)	0.002	1.21 (0.13)	0.07	0.103
**Iron** **(mg/d)**	10.9 (7.5, 15.7)	10 (5.6, 15.3)	11.1 (8.1, 15.2)	11.6 (8.4, 18.4)	1.09 (0.04)	0.028	1.10 (0.04)	0.02	0.023
**Zinc** **(mg/d)**	8.1 (6, 10.9)	8 (5.4, 10.4)	8.1 (6.1, 10.8)	8.2 (6.1, 11.2)	1.05 (0.07)	0.47	1.02 (0.07)	0.751	0.769
**Copper (μg/d)**	707.7 (525.1, 960.8)	609.3 (456.4, 881.3)	733.4 (586, 971.2)	741.6 (541, 1010.9)	1.20 (0.07)	0.002	1.19 (0.07)	0.003	0.005
**Selenium (μg/d)**	69.2 (50.1, 97.6)	59.1 (40.9, 93.2)	71.1 (52.9, 100.1)	77.2 (53.2, 99.7)	1.07 (0.04)	0.039	1.04 (0.04)	0.271	0.307
**Potassium (mg/d)**	1935. 8 (1526.8, 2456.2)	1952.8 (1535.2, 2575.8)	2014.2 (1646.8, 2503.3)	1799.4 (1397.5, 2307)	0.96 (0.18)	0.825	0.64 (0.12)	0.02	0.021
**Sodium** **(mg/d)**	2028.4 (1421.2, 2897.8)	1640.5 (1059, 2264.9)	2121 (1451.4, 2826.4	2484.8 (1818.3, 3240.8)	1.14 (0.05)	0.004	1.15 (0.05)	0.004	0.005

^a^ All values refer to medians; IQR: interquartile range; ^b^ adjusted for age (years), sex, and screen time (hrs). *p* ≤ 0.05 for linear trend across tertiles of ultra-processed food consumption.

## Data Availability

The data presented in this study are available upon reasonable request from the corresponding author.

## Supplementary Materials

The following supporting information can be downloaded at: https://www.mdpi.com/article/10.3390/nu18020272/s1, Table S1: NOVA 4 Food Group derivation with types of food and beverages included in each group.

## Abbreviations

The following abbreviations are used in this manuscript:

UPF	Ultra-processed food
NCD	Non-communicable diseases
HNNHS	National Nutrition and Health Survey
TEI	Total daily energy intake
EAR	Estimated Average Requirement
AI	Acceptable Intake
IOM	Institute of Medicine
SFA	Saturated fatty acids
PUFA	Polyunsaturated fatty acids
