# Supplementary material for: Associations Between Consumption of Ultra-Processed Foods and Diet Quality Among Children and Adolescents
Source: Nutrients. 2026 Jan 14;18(2):272. doi: 10.3390/nu18020272 (PMC12845266; doi:10.3390/nu18020272)
Supplement: Supplementary file 1 [file nutrients-18-00272-s001.zip › nutrients-4027353-supplementary.pdf]

Tables

Table S1. NOVA 4 Food Group derivation with types of food and beverages included in each group.

NOVA 4 Subgroups	Foods and Beverages included in each subgroup
**Ready to eat/heat dishes**	
Ready-to-eat/heat sandwiches	Hotdogs, wheat Arabic pita, crepes, baguettes, sandwiches, tortillas, toast
Ready-to-eat/heat pizza	Pizza
Savoury pies/tarts	Pies (cheese, ham, sausage, vegetables)
Other ready-to-eat/heat-mixed dishes	Stuffed pasta, Greek meat or poultry (souvlaki), meat products (schnitzel, gyros)
**Flavoured dairies products**	
Milk	Condensed sweetened milk
Yoghurt	Flavoured yoghurt
Flavoured dairy drinks	Milkshake, hot chocolate
**Sugar-sweetened beverages**	
Sugar-sweetened and diet soft drinks	Sugar-sweetened and diet soda, iced tea
Fruit drinks	Fruit juices with added sugars or concentrated fruit remixed with water
**Branded breads**	
Grain products	Sliced bread

Pita breads	Arabic pita, pita bread with additives, tortillas, sesame bread rings
**Savoury snacks and baked goods**	
Savoury snacks	Salted crackers, breadsticks, rice cakes, sesame bread rings with cheese, Cheetos, chips (potato, tortillas), popcorn, bread with cheese
Baked goods	Butter croissant, short & puff pastry savoury pies (e.g. cheese pie, spinach pie, meat pie, etc)
**Sweet grain products**	
Bakery sweet products	Chocolate croissants, short & puff pastry sweet pies, ‘tsoureki’, baklava, ‘melomakarono’
Cereal bars and biscuits	Bars, biscuits
Waffles and crepes	Waffles, crepes
Breakfast cereals	Cereals (wheat, whole grain, oat) with added sugar
**Sweets**	
Desserts	Gelatin desserts, traditional sweets with sirup, sweets with chocolate, puddings (chocolate, vanilla), mille-feuille, loukoumi, cakes, cheesecake, profiterole, ice-creams, halva (sesame), Honey sesame bars, chocolate-pie

Sweet pies and tarts	Pies (lemon pie), sweet tarts
Candies & chocolate bars	Candies, jellies, chewing gums, chocolate, chocolate waffle bars, syrups, jams
Other sweet UPFs	Sweet spreads (praline, sesame, peanut butter)
**Other**	
Fast-food or reconstituted meat, poultry, and fish products	Ham from reconstituted meat or poultry, bacon, meat (pate), sausages, nuggets (chicken, cheese)
Fast food or pre-prepared potato products	Fast food, pre-prepared, frozen French fries
Fats, spreads and sauces	Margarine (with or without butter), cheese spread, sauces, dips, cream cheese
Other UPFs	Distilled alcoholic drinks, sparking water, chocolate powder, baby formulas (milk, creams, chamomile formula)
